# The importance of meeting physical activity recommendations and limiting sedentary time in adult survivors of childhood acute lymphoblastic leukemia – associations with fatigue and HRQoL

**DOI:** 10.1007/s11764-026-02027-z

**Published:** 2026-04-30

**Authors:** Laura Jess, Katarina Aili, Jens M. Nygren, Maria Bäck, Marianne Jarfelt

**Affiliations:** 1 Department of Oncology, Institute of Clinical Sciences, Sahlgrenska Academy, University of Gothenburg, Gothenburg, Sweden; 2 Närhälsan Bollebygd Rehabilitation Clinic, Bollebygd, Sweden; 3 Department of Health and Sport, School of Health and Welfare, Halmstad University, Halmstad, Sweden; 4 Department of Health and Nursing, School of Health and Welfare, Halmstad University, Halmstad, Sweden; 5 Department of Occupational Therapy and Physiotherapy, Sahlgrenska University Hospital, Gothenburg, Sweden; 6 Department of Molecular and Clinical Medicine, Institute of Medicine, Sahlgrenska Academy, University of Gothenburg, Gothenburg, Sweden; 7 The Long-Term Follow-Up Clinic for Adult Childhood Cancer Survivors and Cancer Rehabilitation, Sahlgrenska University Hospital, Gothenburg, Sweden

**Keywords:** Adult childhood cancer survivors, MFI-20, Self-reported health outcomes, SF-36, Surveys, Survivorship

## Abstract

**Purpose:**

The aim was to investigate physical activity levels and associations between physical activity levels, sedentary time, fatigue, and health-related quality of life (HRQoL) in adult survivors of childhood acute lymphoblastic leukemia (ALL).

**Method:**

This national cross-sectional study included 426 adult survivors of childhood ALL (diagnosed 1985–2007, 49% women, median age 32 [min–max 19–49]), who completed a questionnaire on physical activity (BHW-Q), sedentary time (SED-GIH-Q), fatigue (MFI-20), and HRQoL (SF-36). Associations were analyzed using linear regression.

**Results:**

Overall, 44% of participants did not meet physical activity recommendations. Median weekly duration of vigorous-intensity and moderate-intensity activity was 45 min (Q1–Q3 15–105) and 120 min (Q1–Q3 45–175), respectively. Median sedentary time was 8 h per day (Q1–Q3 5–11). Not meeting recommendations was significantly associated with greater fatigue (total MFI-20 score β = 1.7, 95% CI 0.1 to 3.3, *p* = 0.033) and lower physical and mental health components of HRQoL (SF-36 PCS β = -3.3, 95% CI -5 to -1.5, *p* < 0.001; SF-36 MCS β = -4.2, 95% CI -6 to -1.8, *p* < 0.001). Sedentary time ≥ 7 h per day was significantly associated with lower mental HRQoL (SF-36 MCS β = -3.6, 95% CI -5.9 to -1.2, *p* = 0.003).

**Conclusion:**

Many adult survivors of childhood ALL had insufficient levels of physical activity and accumulated considerable sedentary time. Insufficient physical activity was associated with greater fatigue and lower HRQoL, while greater sedentary time was associated with lower mental HRQoL. However, given the cross-sectional design, causality cannot be inferred.

**Implications for Cancer Survivors:**

Physical activity promotion is a potential strategy to reduce fatigue and enhance HRQoL among adult survivors of childhood ALL.

**Supplementary Information:**

The online version contains supplementary material available at https://doi.org/10.1007/s11764-026-02027-z.

## Background

Advances in treatment have increased the 5-year survival rate for childhood acute lymphoblastic leukemia (ALL) to over 90% [1]. In Sweden, there are approximately 3000 survivors of childhood ALL. Despite improvements in survival outcomes, a significant number of individuals are at an increased risk of late effects, including secondary malignancies, cardiovascular disease, mental health problems, and fatigue, which significantly impair health-related quality of life (HRQoL) [2–4]. These risks underscore the importance of adopting healthy lifestyle habits, particularly regular physical activity and limiting sedentary time.

Adult survivors of childhood ALL are recommended to follow the World Health Organization’s physical activity recommendations for the general population [5, 6], including 150–300 min of moderate-intensity cardiorespiratory physical activity (MPA) and 75–150 min of vigorous-intensity physical activity (VPA), or a combination thereof, per week. These activities should be combined with strength exercises performed at least two days per week, and a reduced amount of sedentary time [5]. Engaging in regular physical activity is of clear importance to prevent and treat noncommunicable diseases, including cardiovascular disease and some cancers, while also supporting mental health and HRQoL [7]. Physical activity interventions are a well-established, evidence-based approach that has consistently been shown to reduce cancer-related fatigue among adult cancer survivors [8]. However, among adult survivors of childhood ALL, the relationship between physical activity and fatigue appears to be less consistent, suggesting potential differences compared with other groups of cancer survivors [9].

To date, there are no specific threshold values for daily sedentary time for cancer survivors, although previous studies in the general population suggest that the risk of all-cause mortality increases more rapidly when sedentary time exceeds 7–9.5 h per day [10]. Prolonged sedentary time, especially when uninterrupted, also increases the risk of cardiovascular disease and poorer mental health [10, 11]. An earlier study of adult survivors of childhood ALL found that increased sedentary time was associated with low lean mass and increased cardiovascular risk [12]. In addition, previous studies have indicated that childhood ALL survivors are insufficiently physically active [13].

At this time, data on physical activity levels, sedentary time, HRQoL, and fatigue in long-term survivors of childhood ALL are limited. Existing studies have typically been performed shortly after treatment and among young individuals. In addition, no studies have yet been carried out in a Swedish context [9, 14]. In Sweden, healthcare is publicly funded, and follow-up care for adult survivors is provided within the tax-financed healthcare system. There are six long-term follow-up clinics for adult survivors of childhood cancer covering all Swedish regions. However, physiotherapists are not consistently integrated into standard survivorship care. Addressing data gaps may help clinicians develop tailored interventions or enhance patient education to improve HRQoL in this population. Therefore, the primary aim of this study was to evaluate whether Swedish long-term adult survivors of childhood ALL meet physical activity recommendations and to assess their sedentary time. The secondary aim was to examine associations between physical activity levels, sedentary time, fatigue, and HRQoL.

## Methods

The data for this study were collected as part of a Swedish national population-based cross-sectional cohort study of physical health and HRQoL in adult survivors of childhood ALL. The protocol for the cohort study and previous findings have been described in detail elsewhere [15, 16]. The study was approved by the Swedish Ethics Review Authority (Dnr 2019–05181) and conducted in accordance with the Strengthening the Reporting of Observational Studies in Epidemiology (STROBE) guidelines (https://www.strobe-statement.org/).

### Participants

Adult childhood-cancer survivors of childhood ALL were identified through the Swedish Childhood Cancer Registry. Inclusion criteria were age ≥ 18 years, diagnosis of ALL before 18 years of age between 1985 and 2007, and the ability to complete self-reported questionnaire data. The starting year of 1985 was set to ensure that reliable and complete data were available from the Swedish Childhood Cancer Registry and to increase the homogeneity in the cohort since the use of cranial radiation was reduced after 1985. The ending year of 2007 was chosen to reduce the risk of duplicate questionnaires, and as patients treated from 2008 onwards have been subject to detailed follow-ups. Exclusion criteria were severe disabilities preventing questionnaire completion, insufficient proficiency in the Swedish language, unknown postal addresses, relocation outside Sweden, or extended residence abroad.

### Procedure

Data was collected between January and August 2021. After identifying eligible individuals from the Swedish Childhood Cancer Registry, contact information was sourced from the Swedish Population Register (SPAR, Statistics Sweden). A letter was sent with details about the study, along with a consent form and a web link to access the questionnaire. Three reminders were subsequently sent. The third reminder also included a paper version of the questionnaire. All participants provided written informed consent before inclusion in this study.

### Measurements

#### Demographic variables

Background data, including sex, age, age at diagnosis, and type of treatment, were collected from the Swedish Childhood Cancer Registry. All participants received chemotherapy, and some additionally underwent bone marrow transplantation or cranial radiation therapy. Sociodemographic characteristics, including home situation, education, work situation, and lifestyle factors, including body mass index (BMI) and smoking habits, were collected from the study questionnaire.

#### Physical activity and sedentary time

Physical activity and sedentary time were assessed by the Swedish National Board of Health and Welfare questions about physical activity (BHW-Q) and the Swedish School of Sport and Health Sciences (GIH) question for assessing sedentary time (SED-GIH-Q), which are validated and widely used in the general Swedish population and have also been specifically validated in adult childhood cancer survivors [17–19]. The BHW-Q includes separate items for VPA (BHW-Q VPA) and MPA (BHW-Q MPA). Participants report their weekly time spent in activities that cause shortness of breath, as well as time spent in moderate-intensity activities such as walking or cycling performed in bouts of at least 10 min. The SED-GIH-Q assesses sedentary time by asking participants to estimate their typical daily sitting duration. All items use predetermined, fixed time-based response categories. Full item wordings are provided in Supplementary file 1. To determine if an individual meets physical activity recommendations, their VPA score was doubled and added to their MPA score. A total score of ≥ 11 indicates that the recommended 150 min per week are met [17]. Descriptive variables for VPA and MPA were derived from categorical weekly minute ranges by assigning midpoints to closed intervals and top coding for open-ended categories (e.g., < 30 min = 15 min, 30–60 min = 45, 60–90 min = 75). For open-ended categories, plausible midpoint values were assigned (e.g., > 120 min = 135 min for VPA and > 300 min = 330 min for MPA). These values were used to calculate medians and quartiles (Q1 and Q3). Sedentary time was derived analogously from daily sitting time categories using midpoint assignment and top coding for open-ended ranges (e.g., 1–3 h = 2; “all day” = 16 h).

#### Fatigue

Fatigue was measured using the Multidimensional Fatigue Inventory-20 (MFI-20), which is well established and validated in adult cancer survivors [20]. The questionnaire includes 20 items across five subscales: general fatigue, physical fatigue, reduced motivation, reduced activity, and mental fatigue. Each item is scored on a 5-point scale, with subscale scores ranging from 4 to 20, where higher scores indicate greater fatigue. A total score encompassing all five dimensions of the MFI-20 (total MFI-20 score) was computed, ranging from 20 to 100, with higher values indicating greater levels of fatigue. For both unadjusted and adjusted linear regression analyses, we used the total MFI-20 score, as it reflects the overall fatigue burden in adult childhood cancer survivors, reduces multiple testing, and facilitates comparability with previous studies.

#### Health-related quality of life

HRQoL was assessed with the 36-Item Short-Form Health Survey (SF-36) questionnaire, a widely used and validated self-reported questionnaire, including in adult survivors of childhood cancer [21]. The SF-36 measures various aspects of a person’s physical and mental well-being and covers eight health domains, including physical functioning, physical role, pain, general health, vitality, social functioning, emotional role, and mental health. Each scale was scored from 0 to 100, where 100 represents the best possible health rating. Additionally, Mental Component Summary (MCS) and Physical Component Summary (PCS) scores were calculated using the standard scoring algorithm, with MCS reflecting vitality, social functioning, emotional role, and mental health, and PCS reflecting physical functioning, physical role, bodily pain, and general health [22]. Higher values indicate better physical or mental health. All subscales were standardized to population-based z-scores, and factor-score coefficients from Ware et al. were applied to generate norm-based t-scores (mean 50, standard deviation [SD] 10) [22]. Domain scores were calculated when more than 50% of the items were completed, and missing items were replaced with the mean value of completed items within that domain.

### Statistical analysis

Statistical analyses were performed using SPSS, version 25.0 (IBM Corp., Armonk, NY, USA). Descriptive statistics were used to present demographic characteristics of the population and physical activity levels, and sedentary time. Following visual inspection of both histograms and residuals, the variables were assumed to be approximately normally distributed, and the homoscedasticity assumption was met; therefore, parametric analyses were performed. Ordinal data and interval data were described with median and 25th–75th percentiles, continuous variables with mean and SD, and nominal data in numbers and percentages. Univariable and adjusted linear regression analyses were conducted to assess associations between not meeting physical activity recommendations, sedentary time, fatigue, and HRQoL Meeting physical activity recommendations (yes/no) and sedentary time (< 7 vs ≥ 7 h/day) were dichotomized, with the ≥ 7 h/day threshold reflecting evidence of rising cardiometabolic and mortality risk [10]. Robustness analyses using an alternative sedentary time cut-off of ≥ 10 h/day are presented in Supplementary file 2. Potential confounding variables were identified through a review of the literature and based on clinical experience. A directed acyclic graph (DAG) was constructed to identify variables that may be associated with both the exposure and the outcome (Supplementary file 3). Adjustments were made for age, age at diagnosis, sex, BMI, treatment modality (bone marrow transplantation or cranial radiation therapy), and educational level as potential confounders. Smoking was excluded from the analysis due to the low number of smokers, while civil status was excluded based on its presumed limited impact on the outcome. To avoid multicollinearity, variables with conceptual and statistical overlap were reviewed. Age at diagnosis and educational level were included, while years since diagnosis and work status were excluded. The adjusted models were run separately for each outcome based on not meeting the recommended physical activity levels and sedentary time. No multiple-testing correction was applied, and all analyses were conducted using complete-case data.

## Results

### Participants characteristics

**Fig. 1:**
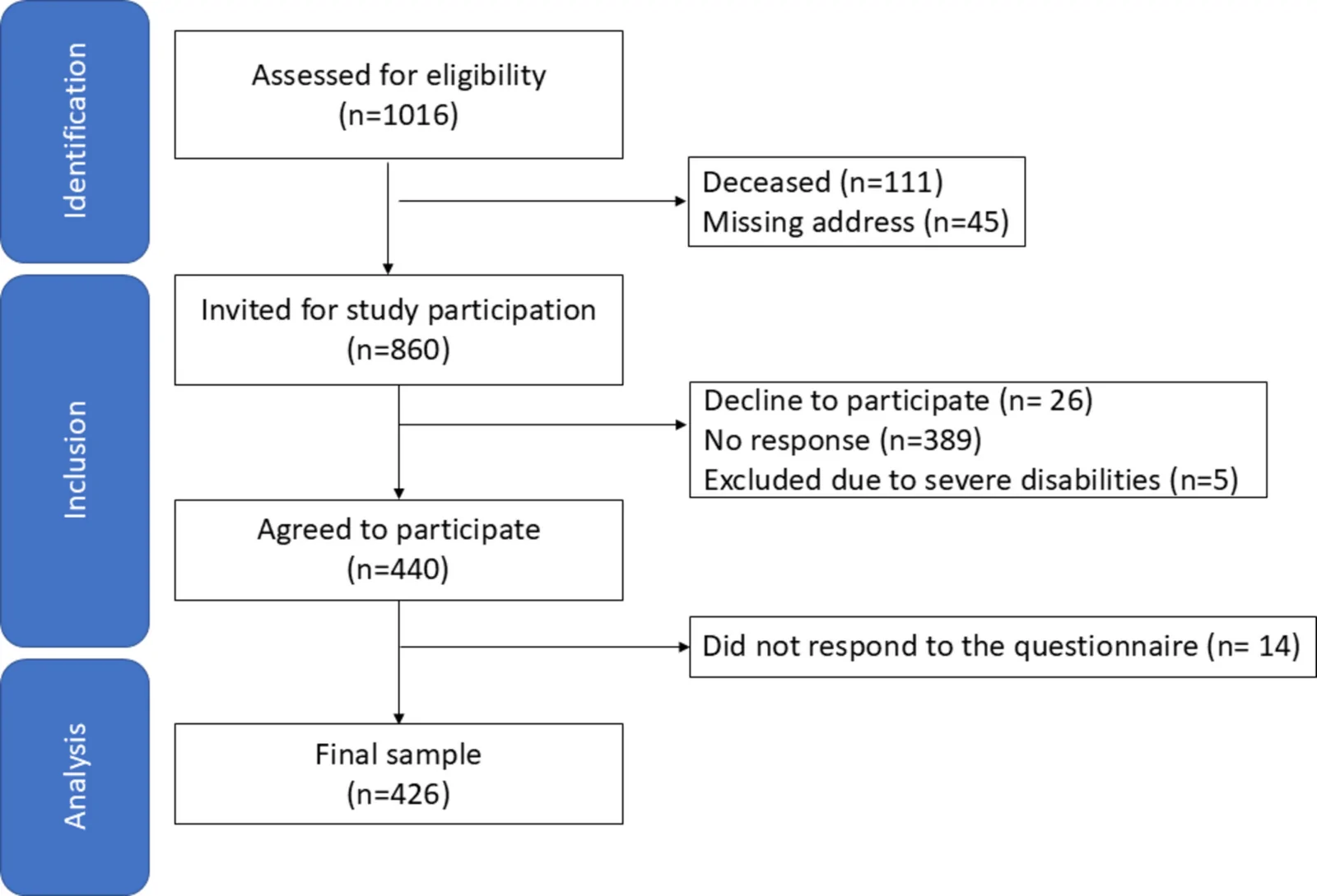
Flowchart of the recruitment procedure

A total of 860 eligible survivors were identified and invited; 440 agreed to participate, and 426 participants were included in the study. The response rate was 49.5%, and all consenting individuals who completed the required questionnaires were included in the analyses. A flow chart of the recruitment procedure is presented in Fig. 1. The median age at participation was 32 (min–max 19–49) years, and at ALL diagnosis was 5 (min–max 0–17) years. Of the participants, 50.9% were men. Demographic characteristics of the study population are presented in Table 1.

**Table 1:** Demographic and clinical characteristics of the study population (*n* = 426)

Adult childhood cancer survivors, *n* = 426^a^	
Sex, women, n (%)	209 (49.1)
Age at diagnosis, years, median (min–max)	5 (0–17)
Years since diagnosis, years, median (min–max)	22 (13–36)
Age at study inclusion, years, median (min–max)	32 (19–49)
Highest level of education, n (%), *n* = 426	
Not completed upper secondary school	18 (4.2)
Completed upper secondary school or higher vocational education	205 (48.1)
University degree	203 (47.6)
Work situation, n (%), *n* = 426	
Employed	276 (64.8)
Studying	76 (17.8)
Long-term sick leave	22 (5.2)
Other	52 (12.3)
Civil status, n (%), *n* = 426	
Married/co-habitation	231 (54.2)
In a relationship	36 (8.5)
Single	159 (37.3)
Treatment, n (%)	
Bone marrow transplantation or cranial radiation therapy, *n* = 426	47 (11)
Chemotherapy, *n* = 426	426 (100)
Lifestyle factors	
Body mass index (kg/m^2^), mean (SD), *n* = 415	25 (4.9)
Smoking, n (%), *n* = 133	26 (6.2)

Abbreviations: *n* number, *SD* standard deviation

^a^Data presented as median (min–max), mean (SD), or as number (n) and percent (%)

### Physical activity level and sedentary time

In total, 44% (*n* = 188) of the participants did not meet current physical activity recommendations. Participants reported a median of 45 (Q1–Q3 15–105) min/week in VPA and a median of 120 (Q1–Q3 45–175) min/week in MPA. Median daily sedentary time was 8 h (Q1–Q3 5–11), with 53% (*n* = 225) of participants reporting sitting for  ≥ 7 h per day. Across participants, 16.2% reported no VPA, 14.2% reported MPA exceeding 300 min/week, and 6.3% were sedentary for basically all day. Distributions of MPA, VPA, and sedentary time are shown in Table 2.

**Table 2:** Self-reported physical activity, sedentary time, health-related quality of life, and fatigue among the participants (*n* = 426)

Variable^a^	
Duration of physical activity (BHW-Q)	
Vigorous physical activity (BHW-Q VPA) (min/week), n/N (%)	
0 min	69/425 (16.2%)
Less than 30 min	88/425 (20.7%)
30–60 min	66/425 (15.5%)
60–90 min	66/425 (15.5%)
90–120 min	52/425 (12.2%)
More than 120 min	84/425 (19.7%)
Median duration of vigorous physical activity (min/week), median (Q1, Q3)	45 (15, 105)
Moderate physical activity (BHW-Q MPA) (min/week), n/N (%)	
0 min	16/425 (3.8%)
Less than 30 min	37/425 (8.7%)
30–60 min	76/425 (17.8%)
60–90 min	59/425 (13.8%)
90–150 min	79/425 (18.5%)
150–300 min	98/425 (23.0%)
More than 300 min	60/425 (14.1%)
Median duration of moderate physical activity (min/week), median (Q1, Q3)	120 (45, 175)
Not meeting physical activity guidelines, n/N (%)	188/425 (44%)
Sedentary time (SED-GIH-Q) (hours/day), n/N (%)	
Basically all day	27/425 (6.3%)
13–15 h,	15/425 (3.5%)
10–12 h	79/425 (18.5%)
7–9 h	104/425 (24.4%)
4–6 h	143/425 (33.6%)
1–3 h	57/425 (13.4%)
Never^b^	0/425 (0%)
Median sedentary time (hours/day), median (Q1, Q3)	8 (5, 11)
Sedentary ≥ 7 h a day, n/N (%)	225/425 (53%)
HRQoL (SF-36 Domains), mean (SD)	
Physical functioning (*n* = 422)	92.4 (15)
Role physical (*n* = 422)	80.1 (33.7)
Bodily pain (*n* = 422)	75.7 (26)
General health (*n* = 422)	64.9 (25)
Vitality (*n* = 422)	51.7 (24.1)
Social functioning (*n* = 421)	72.4 (21.4)
Role emotional (*n* = 422)	71 (39.7)
Mental health (*n* = 422)	68 (21.3)
SF-36 PCS (I = 422)	51.9 (9.1)
SF-36 MCS (*n* = 422)	44.6 (12.3)
Fatigue (MFI-20 Subscales), mean (SD)	
General fatigue (*n* = 407)	11.2 (2.1)
Physical fatigue (*n* = 406)	12.1 (2.1)
Reduced activity (*n* = 407)	12.9 (2.5)
Reduced motivation (*n* = 407)	12.1 (2.7)
Mental fatigue (*n* = 407)	10.5 (2.2)
Total MFI-20 score (*n* = 406)	58.7 (7.8)

Abbreviations: *BHW-Q* The Swedish National Board of Health and Welfare questions about physical activity. *BHW-Q VPA* The Swedish National Board of Health and Welfare question for measuring vigorous physical activity. *BHW-Q MPA* The Swedish National Board of Health and Welfare for measuring moderate physical activity (10-min bouts). *SED-GIH-Q* The Swedish School of Sport and Health Sciences question for assessing sedentary time. *SF-36* Short-Form Health Survey 36 (measuring health-related quality of life on a scale ranging from 0 to 100; higher values indicate better health-related quality of life), *SF-36 PCS* Short-Form Health Survey 36 Physical Component Summary (score based on physical health domains), *SF-36 MCS* Short-Form Health Survey 36 Mental Component Summary (score based on mental health domains), *MFI-20* Multidimensional Fatigue Inventory-20 (measures dimensions of fatigue on a scale ranging from 4–20, higher values indicate higher levels of fatigue; total score ranges from 20–100

^a^Data are presented as number/total number (percent), mean (SD), or median (Q1, Q3)

^b^This category has been retained to reflect the full set of response options included in the original questionnaire

### Health-related quality of life and fatigue

The mean SF-36 PCS score was 51.9 (SD 9.1), the mean SF-36 MCS score was 44.6 (SD 12.3), and the mean total MFI-20 score was 58.7 (SD 7.8) (Table 2).

### Unadjusted and adjusted linear regression

Not meeting physical activity recommendations was associated with significantly poorer physical and mental components of HRQoL (SF-36 PCS; β = −4.6, 95% CI −6.3 to −2.9, *p* < 0.001, and SF-36 MCS; β = −4.3, 95% CI −6.6 to −2, *p* < 0.001). Adult survivors of childhood ALL who did not meet physical activity recommendations reported significantly higher fatigue levels (total MFI-20 score; β = 2, 95% CI 0.5 to 3.5, *p* = 0.01) compared with those who met the recommendations. Being sedentary for ≥ 7 h per day was associated with a significantly lower SF-36 MCS score (β = −3.4, 95% CI −5.8 to −1.1, *p* = 0.004). No significant association was found between sedentary time and the SF-36 PCS score or total MFI-20 score. In adjusted linear regression controlling for age, age at diagnosis, sex, treatment, BMI, and education level, not meeting physical activity recommendations remained significantly associated with lower HRQoL and greater fatigue levels. Not meeting physical activity recommendations was associated with a significantly lower SF-36 PCS score (β = −3.3, 95% CI −5 to −1.5, *p* < 0.001), SF-36 MCS (β = −4.2, 95% CI −6.6 to −1.8, *p* < 0.001), and a significantly higher total MFI-20 score (β = 1.7, 95% CI 0.1 to 3.3, *p* = 0.033). Being sedentary for ≥ 7 h per day was associated with a significantly lower SF-36 MCS score (β = −3.6, 95% CI −5.9 to −1.2, *p* = 0.003). No significant association was found between sedentary time and the SF-36 PCS score or the total MFI-20 score. For a more detailed overview, see Tables 3 and 4 and Supplementary file 4.

**Table 3:** Linear regression analysis of not meeting physical activity recommendations and its association with HRQoL (SF-36 MCS & PCS) and fatigue (Total MFI-20 score)

Dependent variable	Mean (SD) n		Beta (95% CI)	p	Adjusted^a^	
	Does not meet the recommendations	Meets the recommendations			Beta (95% CI)	p
SF-36 PCS	49.3 (10.3) *n* = 186	53.9 (7.5) *n* = 236	−4.6 (−6.3 to −2.9) *n* = 422	< 0.001	−3.3 (−5 to −1.5) *n* = 411	< 0.001
SF-36 MCS	42.3 (13.1) *n* = 186	46.6 (11.3) *n* = 236	−4.3 (−6.6 to −2) *n* = 422	< 0.001	−4.2 (−6.6 to −1.8) *n* = 411	< 0.001
Total MFI-20 score	59.8 (7.7) *n* = 181	57.8 (7.9) *n* = 225	2 (0.5 to 3.5) *n* = 406	0.01	1.7 (0.1 to 3.3) *n* = 395	0.033

Abbreviations: *HRQoL,* health-related quality of life, *SF-36 PCS* Short-Form Health Survey 36 Physical Component Summary, *SF-36 MCS* Short-Form Health Survey 36 Mental Component Summary, *MFI-20* Multidimensional Fatigue Inventory-20, *SD* standard deviation, *CI* confidence interval

^a^Adjusted for age, age at diagnosis, sex, body mass index (BMI), education, and bone marrow transplantation or cranial radiation therapy (BMT/CRT)

**Table 4:** Linear regression analysis of sedentary time  ≥ 7 h and its association with HRQoL (SF-36 MCS & PCS) and fatigue (Total MFI-20 score)

Dependent variable	Mean (SD) n		Beta (95% CI)	p	Adjusted^a^	
	≥ 7 h	< 7 h			Beta (95% CI)	p
SF-36 PCS	51.6 (9.2) *n* = 223	52.1 (9) *n* = 199	−0.4 (−2.2 to 1.3) *n* = 422	0.636	−1 (−2.7 to 0.7) *n* = 411	0.260
SF-36 MCS	43.1 (12.8) *n* = 223	46.5 (11.4) *n* = 199	−3.4 (−5.8 to −1.1) *n* = 422	0.004	−3.6 (−5.9 to −1.2) *n* = 411	0.003
Total MFI-20 score	58.9 (7.5) *n* = 214	58.5 (8.2) *n* = 192	0.5 (−1.1 to 2) *n* = 406	0.537	0.9 (−0.7 to 2.5) *n* = 395	0.253

Abbreviations: *HRQoL* health-related quality of life, *SF-36 PCS* Short-Form Health Survey 36 Physical Component Summary, *SF-36 MCS* Short-Form Health Survey 36 Mental Component Summary, *MFI-20* Multidimensional Fatigue Inventory-20, *SD* standard deviation, *CI* confidence interval

^a^Adjusted for age, age at diagnosis, sex, body mass index (BMI), education, and bone marrow transplantation or cranial radiation therapy (BMT/CRT)

## Discussion

This study contributes to the growing body of research on lifestyle factors in adult survivors of childhood ALL by examining the extent to which participants met physical activity recommendations, their sedentary time, and how these factors were associated with HRQoL and fatigue in a Swedish national cohort. In contrast to most existing research, which mainly examined younger individuals shortly after treatment, this study included adult survivors of childhood ALL 13–36 years after treatment. Only 44% of participants met current physical activity recommendations, and 53% were sedentary for ≥ 7 h each day. Not meeting physical activity recommendations was significantly associated with reduced HRQoL and increased fatigue levels, while prolonged sedentary time was significantly associated with a lower mental component of HRQoL. Our findings suggest that assessing physical activity and sedentary time may be important when developing strategies to address fatigue and improve HRQoL in this population.

The proportion of participants meeting physical activity recommendations was markedly lower than that in the general Swedish population aged 16–84 years (67%) during the same period [23]. This finding aligns with evidence from other countries, indicating that adult survivors of childhood ALL are less likely than the general population to achieve physical activity recommendations [13]. A large U.S. cohort of 2,648 adult survivors of childhood ALL reported a 44% lower likelihood of meeting recommendations [24], while a Canadian study found that only 40% of those ≥ 18 years of age and only 16% of those < 18 years of age met physical activity recommendations [25]. Lower physical activity levels among adult survivors of childhood ALL may be explained by several unique barriers. Prior research has identified treatment-related late effects, such as pain and reduced muscular strength, low self-efficacy, negative peer comparison, and lack of tailored guidance and social or environmental support as factors limiting participation in physical activity [26–28]. Addressing these barriers is important for supporting physical activity participation in this population.

A substantial proportion of participants reported sedentary time of ≥ 7 h per day, a level similar to that observed in the general Swedish population [29]. This finding is consistent with results from the St. Jude Lifetime Cohort study, where adult survivors of childhood ALL were found to spend, on average, 66% of their day being sedentary, which was relatively similar to a proportion of 60% in the general US population [12]. One possible explanation is that adult survivors of childhood ALL may exhibit similar inactivity patterns to adults in the general population due to shared environmental and social factors. However, as sedentary time is recognized as an independent risk factor for adverse health outcomes regardless of physical activity levels, further studies are needed to understand the prevalence and the impact of sedentary behavior in this population.

In this cohort, not meeting physical activity recommendations was associated with lower physical and mental components of HRQoL. This aligns with a prior study showing that higher levels of physical activity were associated with better HRQoL among adult childhood cancer survivors [30]. There is growing evidence that the relationship between physical activity and mental health is bidirectional, where increased physical activity reduces symptoms of mental health, but poorer mental health also predicts lower physical activity [31]. Given this, interventions aiming to promote physical activity should also address psychological barriers and aim to concurrently improve mental health and avoid reinforcing cycles of physical inactivity and distress.

The observed association between not meeting physical activity recommendations and higher fatigue levels in this cohort is supported by a systematic review, which found that increased physical activity is generally associated with lower levels of fatigue in childhood cancer survivors [9]. However, the quality of evidence remains a concern due to small sample sizes, variability in interventions, and inconsistencies in fatigue measurement and diagnosis definitions.

A surprising finding was that being sedentary for ≥ 7 h per day was only significantly associated with the mental component of HRQoL. This contrasts with findings from previous research in the general population and among adult cancer survivors, where reduced sedentary time has been modestly associated with improved physical HRQoL, while links with the mental component of HRQoL have been inconsistent or non-significant [32, 33]. One possible explanation is that adult survivors of childhood cancer may experience long-term psychological effects that make their mental HRQoL more sensitive to sedentary behavior. Nevertheless, to better understand the underlying mechanisms of this relationship and to address the evident knowledge gap, further studies using longitudinal designs, randomized clinical trials, or objective measurement methods are needed.

## Strengths and limitations

Population-based studies are ideal for estimating how common a condition or behavior is. A major strength of this study is its use of a national cohort, in which all individuals diagnosed with ALL in Sweden during a defined period (1985–2007) were invited to participate. Another strength is that the questions used to assess physical activity and sedentary time were previously validated against accelerometer data in this population [19]. Also, the SF-36 has been specifically validated in this population, and the MFI-20 has been validated among adult cancer survivors.

An important limitation was that the response rate to the questionnaire (49.5%) was below the commonly accepted minimum of 60%, which may affect the validity and generalizability of the findings. To examine the potential impact of this limitation, a non-respondent analysis was performed. The non-responders did not differ from responders with respect to age, age at diagnosis, or treatment modality. However, a higher proportion of women participated in the study compared with those who did not respond (48.8% vs 40%) [15]. Non-response bias is possible, as individuals experiencing greater fatigue or more sedentary lifestyles may have been less likely to participate. If so, physical activity levels in the analytic sample may be slightly overestimated, and fatigue levels may be underestimated. This possibility should be considered when interpreting generalizability. The use of self-reported data is another limitation because it introduces the possibility of recall bias. Participants substantially overestimate moderate-to-vigorous physical activity by tens of minutes and underestimate sedentary time by approximately 1–2 h/day [34, 35]. Ideally, physical activity should be objectively measured, although, as noted, the questions on physical activity and sedentary time have been validated against accelerometer data in this population [19]. Self-report instruments remain appropriate for larger populations, given feasibility considerations, low respondent burden, and consistency across survey cycles [36].

Notably, the associations between not meeting physical activity recommendations and reduced HRQoL and increased fatigue levels, as well as between prolonged sedentary time and mental HRQoL, were statistically significant but weak. Larger studies are needed to confirm and further explore these findings with respect to clinical relevance. The analysis was adjusted for relevant confounders, which strengthens the validity of the observed associations; however, the possibility of residual confounding from unmeasured factors cannot be excluded. Moreover, the cross-sectional design limits causal inferences between physical activity, sedentary behavior, HRQoL, and fatigue. Longitudinal and interventional studies are needed to clarify the causal relationship between the self-reported outcomes in adult survivors of childhood ALL. Although causality cannot be established in this work, the cross-sectional design provides valuable insights into associations that can support clinical decisions and help prioritize areas for further research.

## Clinical implications

Routine screening of physical activity and sedentary time using quick assessment tools may help clinicians identify survivors in need of targeted support. Providing tailored advice or referral to physiotherapists or exercise specialists, together with brief physical activity counseling during long-term follow-up visits, including goal setting and individualized activity prescriptions, may enhance engagement in physical activity and help survivors reach recommended levels of MPA and VPA. Simple strategies to reduce sedentary time, such as prompts for brief movement every 30 to 60 min, are feasible to implement for most survivors.

## Conclusion

In this Swedish national cross-sectional cohort study, less than half of the adult survivors of childhood ALL met physical activity recommendations, and their sedentary time was comparable to that of the general Swedish population. A significant association was identified between not meeting physical activity recommendations and both fatigue and HRQoL, as well as between sedentary time and the mental component of HRQoL. These results further emphasize the significance of supporting physical activity as a potential strategy to improve health-related outcomes in adult survivors of childhood ALL. However, randomized and longitudinal studies are needed to establish causality.

## Supplementary Information

Below is the link to the electronic supplementary material.

ESM 1 (DOCX 14.0 KB)

ESM 2 (DOCX 19.0 KB)

ESM 3 (TIFF 4.20 MB)

ESM 4 (DOCX 40.0 KB)

## Data Availability

Data from the current study are not publicly available due to ethical conditions, but are available from the corresponding author upon reasonable request.

## Abbreviations

ALL: Acute lymphoblastic leukemia
BHW-Q: Swedish National Board of Health and Welfare questions for physical activity
BHW-Q MPA: Swedish National Board of Health and Welfare question for measuring moderate physical activity
BHW-Q VPA: Swedish National Board of Health and Welfare question measuring vigorous physical activity
CI: Confidence interval
DAG: Directed acyclic graph
HRQoL: Health-related quality of life
MCS: Mental Component Summary
MFI-20: Multidimensional Fatigue Inventory-20
MPA: Moderate physical activity
PCS: Physical Component Summary
SD: Standard deviation
SED-GIH-Q: Swedish School of Sport and Health Sciences question for assessing sedentary time
SF-36: 36-Item Short-Form Health Survey
VPA: Vigorous physical activity
