# Supplementary material for: The importance of meeting physical activity recommendations and limiting sedentary time in adult survivors of childhood acute lymphoblastic leukemia – associations with fatigue and HRQoL
Source: J Cancer Surviv. 2026 Apr 30;20(Suppl 2):1916–26. doi: 10.1007/s11764-026-02027-z (PMC13744527; doi:10.1007/s11764-026-02027-z)
Supplement: Supplementary file 1 [file 11764_2026_2027_MOESM1_ESM.docx]

**Supplementary file 1 - Physical activity and sedentary time questions**

The BHW-Q contains two questions, one for measuring vigorous physical activity (BHW-Q VPA) and one for measuring moderate physical activity (BHW-Q MPA).

Vigorous physical activity (VPA) was assessed by the question “During a regular week, how much time do you spend exercising at a level that makes you short of breath, for example running, fitness class, or ball games?”, with the following response categories: 0 minutes (1), less than 30 minutes (2), 31–60 minutes (3), 61–90 minutes (4), 91–120 minutes (5), more than 120 minutes (6).

Moderate physical activity (MPA) was assessed by the question “During a regular week, how much time are you physically active in ways that are not exercise, for example, walks, bicycling, or gardening? Add together all activities lasting at least 10 min.”, with the following response categories: 0 minutes (1), less than 30 minutes (2), 31–60 minutes (3), 61–90 minutes (4), 91–150 minutes (5), 151–300 minutes (6), and more than 300 minutes (7).

The SED-GIH-Q contains one question and sedentary time was assessed by the question “How much time do you spend sitting during a normal day, not counting sleep?”, with the following response categories: basically, all day, 13–15 hours, 10–12 hours, 7–9 hours, 4–6 hours, 1–3 hours, and never.
