# Supplementary material for: The importance of meeting physical activity recommendations and limiting sedentary time in adult survivors of childhood acute lymphoblastic leukemia – associations with fatigue and HRQoL
Source: J Cancer Surviv. 2026 Apr 30;20(Suppl 2):1916–26. doi: 10.1007/s11764-026-02027-z (PMC13744527; doi:10.1007/s11764-026-02027-z)
Supplement: Supplementary file 2 [file 11764_2026_2027_MOESM2_ESM.docx]

**Supplementary file 2**

Results from linear regression analysis of sedentary time ≥ 10 hours and its association with HRQoL (SF-36 MCS & PCS) and fatigue (Total MFI-20 score)

|  | Mean (SD)  n | |  |  |
| --- | --- | --- | --- | --- |
| Dependent variable | ≥10 hours | <10 hours | Beta (95% CI) | p |
|  |  |  |  |  |
| SF-36 PCS | 51.7 (9.6)  n=120 | 51.9 (8.9)  n=302 | -0.1 (-2.1 to 1.8)  n=422 | 0.893 |
| SF-36 MCS | 45.5 (11.7) n=120 | 42.6 (13.3) n=302 | -2.9 (-5.5 to -0.4)  n=422 | 0.026 |
| Total MFI-20 score | 58.54 (8.1) n=114 | 59.1 (7.2) n=292 | 0.6 (-1.1 to 2.3) n=406 | 0.500 |
|  |  |  |  |  |

Abbreviations: *HRQoL* health-related quality of life, *SF-36 PCS* Short-Form Health Survey 36 Physical Component Summary, *SF-36 MCS* Short-Form Health Survey 36 Mental Component Summary, *MFI-20* Multidimensional Fatigue Inventory-20, *SD* standard deviation, *CI* confidence interval.
