# Supplementary material for: The importance of meeting physical activity recommendations and limiting sedentary time in adult survivors of childhood acute lymphoblastic leukemia – associations with fatigue and HRQoL
Source: J Cancer Surviv. 2026 Apr 30;20(Suppl 2):1916–26. doi: 10.1007/s11764-026-02027-z (PMC13744527; doi:10.1007/s11764-026-02027-z)
Supplement: Supplementary file 3 [file 11764_2026_2027_MOESM4_ESM.docx]

**Supplementary file 4- Adjusted models including key covariates**

Table1a. Results from multivariable linear regression of *SF-36 PCS (dependent variable)* with *not meeting physical activity recommendations* and adjustment factors (SPSS output)

| Model | | Unstandardized Coefficients | | Standardized Coefficients | t | Sig. | 95.0% Confidence Interval for B | |
| --- | --- | --- | --- | --- | --- | --- | --- | --- |
|  |  | B | Std. Error | Beta |  |  | Lower Bound | Upper Bound |
| 1 | (Constant) | 53.555 | 3.326 |  | 16.102 | <.001 | 47.017 | 60.094 |
|  | Does not meet the PA recommendations^a^ | -3.261 | .873 | -.178 | -3.734 | <.001 | -4.978 | -1.544 |
|  | Age^b^ | -.187 | .065 | -.158 | -2.896 | .004 | -.314 | -.060 |
|  | Age at diagnosis^c^ | -.058 | .110 | -.028 | -.524 | .601 | -.274 | .159 |
|  | Sex^d^ | 2.405 | .868 | .133 | 2.772 | .006 | .699 | 4.111 |
|  | BMI^e^ | -.132 | .091 | -.071 | -1.453 | .147 | -.310 | .046 |
|  | Education^f^ | 2.512 | .771 | .158 | 3.258 | .001 | .996 | 4.027 |
|  | BMT/CRT^g^ | -4.544 | 1.398 | -.156 | -3.249 | .001 | -7.292 | -1.795 |
|  | | | | | | | | |

^a^0 = Does not meet the recommendations, 1 = Meets the recommendations.

^b^Age (in years).

^c^Age at diagnosis (in years).

^d^1 = female, 2 = male.

^e^ Body mass index (kg/m^2^).

^f^1 = Not completed upper secondary school, 2 = Completed upper secondary school or higher vocational education, 3 = University degree.

^g^0= no, 1=yes

Abbreviations: *SF-36 PCS* Short-Form Health Survey 36 Physical Component Summary, *B* beta, *Std. Error* standard error, *t* t-statistic, *Sig.* significance (p-value), *PA* physical activity, *BMI* body mass index, *BMT/CRT* bone marrow transplantation or cranial radiation therapy.

Table 1b Results from multivariable linear regression of *SF-36 MCS* (dependent variable) with *not meeting physical activity recommendations* and adjustment factors (SPSS output)

| Model | | Unstandardized Coefficients | | Standardized Coefficients | t | Sig. | 95.0% Confidence Interval for B | |
| --- | --- | --- | --- | --- | --- | --- | --- | --- |
|  |  | B | Std. Error | Beta |  |  | Lower Bound | Upper Bound |
| 1 | (Constant) | 36.196 | 4.636 |  | 7.808 | <.001 | 27.083 | 45.309 |
|  | Does not meet the PA recommendations^a^ | -4.214 | 1.217 | -.172 | -3.462 | <.001 | -6.607 | -1.821 |
|  | Age^b^ | .180 | .090 | .113 | 1.999 | .046 | .003 | .357 |
|  | Age at diagnosis^c^ | -.045 | .154 | -.016 | -.292 | .770 | -.347 | .257 |
|  | Sex^d^ | 4.536 | 1.209 | .186 | 3.751 | <.001 | 2.159 | 6.914 |
|  | BMI^e^ | -.083 | .126 | -.033 | -.658 | .511 | -.331 | .165 |
|  | Education^f^ | .169 | 1.075 | .008 | .157 | .875 | -1.943 | 2.281 |
|  | BMT/CNSI^g^ | -.684 | 1.949 | -.018 | -.351 | .726 | -4.515 | 3.147 |
|  | | | | | | | | |

^a^0 = Does not meet the recommendations, 1 = Meets the recommendations.

^b^Age (in years).

^c^Age at diagnosis (in years).

^d^1 = female, 2 = male.

^e^ Body mass index (kg/m^2^).

^f^1 = Not completed upper secondary school, 2 = Completed upper secondary school or higher vocational education, 3 = University degree.

^g^0= no, 1=yes

Abbreviations: *SF-36 PCS* Short-Form Health Survey 36 Physical Component Summary, *B* beta, *Std. Error* standard error, *t* t-statistic, *Sig.* significance (p-value), *PA* physical activity, *BMI* body mass index, *BMT/CRT* bone marrow transplantation or cranial radiation therapy.

Table 1c Results from multivariable linear regression of *total MFI-20 score* (dependent variable) with *not meeting physical activity recommendations* and adjustment factors (SPSS output)

| Model | | Unstandardized Coefficients | | Standardized Coefficients | t | Sig. | 95.0% Confidence Interval for B | |
| --- | --- | --- | --- | --- | --- | --- | --- | --- |
|  |  | B | Std. Error | Beta |  |  | Lower Bound | Upper Bound |
| 1 | (Constant) | 56.816 | 3.171 |  | 17.915 | <.001 | 50.581 | 63.052 |
|  | Does not meet the PA recommendations^b^ | 1.744 | .814 | .110 | 2.143 | .033 | .144 | 3.344 |
|  | Age^c^ | .133 | .060 | .128 | 2.208 | .028 | .015 | .251 |
|  | Age at diagnosis^d^ | .036 | .102 | .020 | .356 | .722 | -.165 | .237 |
|  | Sex^e^ | -1.721 | .812 | -.109 | -2.119 | .035 | -3.317 | -.125 |
|  | BMI^f^ | -.049 | .084 | -.031 | -.584 | .559 | -.214 | .116 |
|  | Highest level of education^g^ | .271 | .726 | .019 | .373 | .709 | -1.156 | 1.698 |
|  | BMT/CNSI^h^ | -.743 | 1.309 | -.029 | -.567 | .571 | -3.316 | 1.831 |
|  | | | | | | | | |

^a^0 = Does not meet the recommendations, 1 = Meets the recommendations.

^b^Age (in years).

^c^Age at diagnosis (in years).

^d^1 = female, 2 = male.

^e^ Body mass index (kg/m^2^).

^f^1 = Not completed upper secondary school, 2 = Completed upper secondary school or higher vocational education, 3 = University degree.

^g^0= no, 1=yes

Abbreviations: *SF-36 PCS* Short-Form Health Survey 36 Physical Component Summary, *B* beta, *Std. Error* standard error, *t* t-statistic, *Sig.* significance (p-value), *PA* physical activity, *BMI* body mass index, *BMT/CRT* bone marrow transplantation or cranial radiation therapy.

Table 2a Results from multivariable linear regression of SF-36 *PCS* (dependent variable) with being *sedentary ≥ 7 hours a day* and adjustment factors (SPSS output)

| Model | | Unstandardized Coefficients | | Standardized Coefficients | t | Sig. | 95.0% Confidence Interval for B | |
| --- | --- | --- | --- | --- | --- | --- | --- | --- |
|  |  | B | Std. Error | Beta |  |  | Lower Bound | Upper Bound |
| 1 | (Constant) | 53.647 | 3.396 |  | 15.798 | <.001 | 46.971 | 60.322 |
|  | Sedentary ≥ 7 hours a day^a^ | -.977 | .866 | -.054 | -1.128 | .260 | -2.678 | .725 |
|  | Age^b^ | -.220 | .066 | -.186 | -3.322 | <.001 | -.351 | -.090 |
|  | Age at diagnosis^c^ | -.054 | .112 | -.026 | -.482 | .630 | -.275 | .166 |
|  | Sex^d^ | 2.775 | .877 | .153 | 3.164 | .002 | 1.051 | 4.500 |
|  | BMI^e^ | -.185 | .091 | -.100 | -2.038 | .042 | -.363 | -.007 |
|  | Highest level of education^f^ | 2.850 | .783 | .179 | 3.641 | <.001 | 1.311 | 4.389 |
|  | BMT/CNSI^g^ | -5.010 | 1.414 | -,172 | -3.544 | <.001 | -7.789 | -2.231 |
|  | | | | | | | | |

^a^ 0 = <7 hours, 1 = ≥7 hours of sedentary time.
^b^Age (in years).

^c^Age at diagnosis (in years).

^d^1 = female, 2 = male.

^e^ Body mass index (kg/m^2^).

^f^1 = Not completed upper secondary school, 2 = Completed upper secondary school or higher vocational education, 3 = University degree.

^g^0= no, 1=yes

Abbreviations: *SF-36 PCS* Short-Form Health Survey 36 Physical Component Summary, *B* beta, *Std. Error* standard error, *t* t-statistic, *Sig.* significance (p-value), *PA* physical activity, *BMI* body mass index, *BMT/CRT* bone marrow transplantation or cranial radiation therapy.

Table 2b Results from multivariable linear regression of SF-36 *MCS* (dependent variable) with being *sedentary ≥ 7 hours a day* and adjustment factors (SPSS output)

| Model | | Unstandardized Coefficients | | Standardized Coefficients | t | Sig. | 95.0% Confidence Interval for B | |
| --- | --- | --- | --- | --- | --- | --- | --- | --- |
|  |  | B | Std. Error | Beta |  |  | Lower Bound | Upper Bound |
| 1 | (Constant) | 37.278 | 4.677 |  | 7.970 | <.001 | 28.083 | 46.473 |
|  | Sedentary ≥ 7 hours a day^a^ | -3.574 | 1.192 | -.147 | -2.998 | .003 | -5.918 | -1.230 |
|  | Age^b^ | .107 | .091 | .067 | 1.167 | .244 | -.073 | .286 |
|  | Age at diagnosis^c^ | -.021 | .155 | -.008 | -.139 | .890 | -.325 | .282 |
|  | Sex^d^ | 5.101 | 1.208 | .209 | 4.222 | <.001 | 2.726 | 7.476 |
|  | BMI^e^ | -.132 | .125 | -.053 | -1.059 | .290 | -.378 | .113 |
|  | Highest level of education^f^ | .790 | 1.078 | .037 | .733 | .464 | -1.329 | 2.910 |
|  | BMT/CNSI^g^ | -1.189 | 1.947 | -.030 | -.611 | .542 | -5.017 | 2.639 |
|  | | | | | | | | |

^a^ 0 = <7 hours, 1 = ≥7 hours of sedentary time.
^b^Age (in years).

^c^Age at diagnosis (in years).

^d^1 = female, 2 = male.

^e^ Body mass index (kg/m^2^).

^f^1 = Not completed upper secondary school, 2 = Completed upper secondary school or higher vocational education, 3 = University degree.

^g^0= no, 1=yes

Abbreviations: *SF-36 PCS* Short-Form Health Survey 36 Physical Component Summary, *B* beta, *Std. Error* standard error, *t* t-statistic, *Sig.* significance (p-value), *PA* physical activity, *BMI* body mass index, *BMT/CRT* bone marrow transplantation or cranial radiation therapy.

Table 2c Results from multivariable linear regression of *total MFI-20 score* (dependent variable) with being *sedentary ≥ 7 hours a day* and adjustment factors (SPSS output)

| Model | | Unstandardized Coefficients | | Standardized Coefficients | t | Sig. | 95.0% Confidence Interval for B | |
| --- | --- | --- | --- | --- | --- | --- | --- | --- |
|  |  | B | Std. Error | Beta |  |  | Lower Bound | Upper Bound |
| 1 | (Constant) | 56.759 | 3.194 |  | 17.769 | <.001 | 50.478 | 63.039 |
|  | Sedentary ≥ 7 hours a day^b^ | .915 | .798 | .058 | 1.146 | .253 | -.655 | 2.485 |
|  | Age^c^ | .155 | .061 | .150 | 2.544 | .011 | .035 | .275 |
|  | Age at diagnosis^d^ | .030 | .103 | .016 | .289 | .773 | -.172 | .232 |
|  | Sex^e^ | -1.935 | .812 | -.123 | -2.383 | .018 | -3.532 | -.338 |
|  | BMI^f^ | -.026 | .083 | -.016 | -.309 | .758 | -.189 | .138 |
|  | Highest level of education^g^ | .032 | .728 | .002 | .044 | .965 | -1.400 | 1.465 |
|  | BMT/CNSI^h^ | -.497 | 1.308 | -.020 | -.380 | .704 | -3.069 | 2.076 |

^a^ 0= <7 hours, 1 = Sedentary time ≥ 7 hours.

^b^Age (in years).

^c^Age at diagnosis (in years).

^d^1 = female, 2 = male.

^e^ Body mass index (kg/m^2^).

^f^1 = Not completed upper secondary school, 2 = Completed upper secondary school or higher vocational education, 3 = University degree.

^g^0= no, 1=yes

Abbreviations: *SF-36 PCS* Short-Form Health Survey 36 Physical Component Summary, *B* beta, *Std. Error* standard error, *t* t-statistic, *Sig.* significance (p-value), *PA* physical activity, *BMI* body mass index, *BMT/CRT* bone marrow transplantation or cranial radiation therapy.
